# Suicidal behaviour amid first wave of COVID-19 pandemic in Malaysia: Data from the COVID-19 mental health international (COMET-G) study

**DOI:** 10.3389/fpsyt.2022.998888

**Published:** 2022-10-18

**Authors:** Salmi Razali, Jo Anne Saw, Nurul Azreen Hashim, Nor Jannah Nasution Raduan, Dina Tukhvatullina, Daria Smirnova, Konstantinos N. Fountoulakis

**Affiliations:** ^1^Department of Psychiatry, Faculty of Medicine, Universiti Teknologi MARA, Sungai Buloh, Selangor, Malaysia; ^2^Institute of Pathology, Laboratory and Forensic Medicine (I-PPerForM), Universiti Teknologi MARA (UiTM), Sungai Buloh, Selangor, Malaysia; ^3^Maternofetal and Embryo Research Group (MatE), Universiti Teknologi MARA (UiTM), Sungai Buloh, Selangor, Malaysia; ^4^Centre for Global Public Health, Institute of Population Health Sciences, Queen Mary University of London, London, United Kingdom; ^5^International Centre for Education and Research in Neuropsychiatry (ICERN), Samara State Medical University, Samara, Russia; ^6^Department of Psychiatry, Narcology, Psychotherapy and Clinical Psychology, Samara State Medical University, Samara, Russia; ^7^3rd Department of Psychiatry, School of Medicine, Aristotle University of Thessaloniki Greece, Thessaloniki, Greece

**Keywords:** suicidal behaviour, COVID-19, Malaysia, mental health, pandemic

## Abstract

During the COVID-19 pandemic, numerous social and life changes were implemented to curb the spread of the disease. The effect of lockdown and isolation predisposes the general population to various psychological health concerns. The existing determinants of suicidal behaviour were further added with social isolation, financial stress, depression, and other pandemic-related stressors. Hence, our study aimed to investigate suicidal behaviour and the associated factors among Malaysians during the COVID-19 pandemic. It is a cross-sectional online questionnaire survey that used convenient sampling, where the survey was disseminated to the public *via* Google Forms through social media during the first wave of the COVID-19 pandemic in Malaysia. This study is also part of a large international COVID-19 mental health international study for the general population (COMET-G). This research revealed concerns about issues related to suicidal behaviours during the beginning of the COVID-19 pandemic. Suicidal behaviours were associated with depression, sex, marital status, educational level, employment type, residential area, number of people living together, number of children, and family dynamics. The pandemic effects from psychological, social, and economic perspectives will definitely take more time for recovery. Future prevention and protection are needed especially for the highly at-risk group on top of the general population in any future unforeseen circumstances of the pandemic.

## Introduction

The COVID-19 infection leaves long-term neuropsychiatric symptoms, including sleep problems, anxiety, depressed mood, and irritability, making them more vulnerable to mental health disturbances. Furthermore, the effects of lockdown, isolation, and quarantine predisposed the general population to psychological and emotional burdens, putting them at risk of mental health disorders (1). Although COVID-19 had its outbreak in China in December 2019, Malaysia only had the first COVID-19 case in February 2020. Due to this threat, Malaysia had its first movement control order (MCO) on 18 March 2020 (2). During this time, travelling across districts and states was to be avoided, and mass gatherings were postponed, especially in religious houses. These continued in a few separate episodes until the end of 2021 (3).

There was heterogeneous nomenclature for definitions of suicidal behaviour according to literature. Suicidal behaviour is defined as suicide attempts, which are self-directed injurious acts with at least some intent to end one’s own life, which range from completed suicide to highly lethal and failed suicide attempts to low-lethality, usually impulsive attempts due to social crisis (4). Factors affecting suicidal behaviour are complex and variable. These factors can generally be divided into sociodemographic, socioeconomic, socio-political, geographical, cultural, lifestyle, and health- or clinical-related factors (5). Personality and individual differences, cognitive factors, social aspects, and adverse life events are the main psychological factors contributing to the suicidal behaviour (6). The Malaysian data collected before the COVID-19 pandemic showed that the determinants of suicidal behaviour were income, age, gender, ethnicity, education, marital status, self-rated health, and being diagnosed with diabetes and hypercholesterolemia (7).

During the COVID-19 pandemic, the existing determinants of suicidal behaviour were added to social isolation, financial stress, depression, limited or variable access to healthcare services, and other pandemic-related stressors (8). Studies done during the COVID-19 pandemic found that suicidal ideation was associated with loneliness, anxiety, depression, insomnia, impaired family functioning, a history of mental health issues, alcohol misuse, COVID-19-related stress symptoms, concerns over COVID-19, having tested positive for COVID-19, a younger age, an unmarried or divorced marital status, living alone, being a military veteran, previous homelessness, financial strain, housing instability, unemployment, poor perceived quality of physical health, disability, and living with an individual with frail health (9–12). A study in Italy showed that 14% of respondents were at higher risk of having suicidal ideation after being unemployed due to the pandemic (13). While another study among healthcare workers (HCW) in Malaysia during the COVID-19 pandemic discovered that suicidal ideation was linked to depression and early career status of less than 10 years in service (14).

Our study investigated suicidal behaviour and the associated factors among Malaysians during the COVID-19 pandemic. This study is part of a large international COVID-19 mental health international study for the general population (COMET-G).

## Methodology

### Study design

This study is part of the global joint project of more than 40 countries worldwide initiated by the Aristotle University of Thessaloniki and the Mental Health Sector of the Scientific Research Institute of the Pan-Hellenic Medical Association, Greece. It is a cross-sectional online questionnaire survey that used convenient sampling.

### Data collection

In Malaysia, the survey was disseminated to the public using Google Forms through social media (e.g., Facebook and Twitter) during the first wave of the COVID-19 pandemic. The distribution of the survey was done from 1 July 2020 (average of six cases per week) to 6 October 2020 (average of 338 cases per week) during the initial wave of the COVID-19 pandemic in Malaysia.

The selection criteria were participants aged 18 years and above, who could read Malay or English, and who had access to the internet to receive the online form. All potential participants were provided with an explanation regarding the risk and benefits of the study on the introductory page of the online questionnaire. Participants who agreed to participate were invited to answer the questionnaires. Implied consent was gathered when the participant proceeded to the next page, where the actual survey began. This study was approved by the local institutional research ethics committee, REC/06/2020 (MR/109).

### Measurement tools

Embedded in the survey was Pro Forma questionnaire for sociodemographic data, including sex, age, marital status, residential areas, educational status, employment, and status of being a HCW status, living condition (including the number of people living together, number of children, and status of living with a vulnerable family member), and their background medical disease. The detailed categorisation of independent variables followed the main COMET-G study, which include sex statuses (female, male, and other/do not want to declare), age (years old) (≤ 21, 22–45, 46–60, and ≥ 61), marital status [single or married (or in a civil partnership), divorced (or estranged), co-habitant, widower, or other], educational level (elementary school or less, high school degree or equivalent, bachelor’s degree, master’s degree, and doctorate), residential area [capital city, city > 1 million population, city (100,000–1 million population), town (20,000–100,000 inhabitants), town (< 20,000 inhabitants), rural area to village and other], number of people living together (one, two, three, four, and five or more), number of children (zero, one, two, three, and four or more), living with vulnerable people (No or Yes), employment status (working at the public sector, working at private sector, self-employed, retired, unemployed, housekeeper, college or university students, not working by choice, and other), status as HCW (doctor, nurse, other HCW with direct contacts to clinical work, administrative employee at a hospital, other hospital staff, and do not work in the health sector), and status of the chronic medical illness (No or Yes).

For mental health status, the presence of depression and anxiety were assessed with the Center for Epidemiologic Studies Depression Scale (CES-D) and the State-Trait Anxiety Inventory (STAI-Y). The English version of these questionnaires was prepared by the lead collaborating centre, while the Malay version was prepared after translation and discussion between local researchers with reference to the Malay version of the questionnaires (15). The total scores of those questionnaires were used to indicate depression when the CES-D score was above the cut-off score of 23/24 and the algorithm score was above 9.03 as per the categorisation in COMET-G (16) and anxiety, respectively. Furthermore, statements assessing the emotional changes (joy and melancholy) were also included in the survey. Spirituality was also assessed using a Likert score ranging from 0 to 3.

Participants were also asked about the family dynamics during the lockdown due to the pandemic. Using Likert scores ranging from “−2 = much less,” “−1 = less,” “0 = same,” “1 = more,” and “2 = much more,” the participants were asked about their needs to communicate with other members of their family, receive emotional support from other members of the family, and the presence of any conflicts with the rest of your family members during the period of lockdown due to the pandemic. They were also asked about changes in the overall quality of relationships with the other members of their family compared to the one before the quarantine by choosing the score: “−2 = much worse,” “−1 = worse,” “0 = it has not changed,” “1 = a little bit better,” and “2 = much better.” Similar scoring was used for the status of the participant’s financial status. Furthermore, a question was also given about managing to maintain a basic daily routine (such as waking up in the morning, regular meals, sleeping hours, and routine activities) both for participants and their families, the scores ranged from “0 = not at all,” “1 = a little,” “2 = most of the times,” and “3 = always.” Finally, the level of difficulty managing their children’s daily life and behaviour was also asked, and the scores ranged from “−2 = much more difficult than before,” “−1 = somehow more difficult but not always,” “0 = same as always (also if the participant does not have children),” “1 = somehow easier but not always,” and “2 = much easier than before.” The overall family dynamics are represented by the total scores of all domains of the family dynamic scores.

Suicidal behaviour was measured with the Risk Assessment Suicidality Scale (RASS) (17). The English version of the survey was prepared by the lead collaborating centre, while the Malay version was prepared after translation and discussion between local researchers. The overall suicidal behaviour was indicated by the total score of RASS, while the suicidal intention, lifetime suicidal behaviour, and history of suicidal behaviour were assessed using the RASS subscales of “intention,” “life,” and “history,” respectively (17). The full protocol can be found elsewhere (16).

### Statistical analyses

Descriptive statistics were calculated for the independent variables: key sociodemographic variables (including sex, age, marital status, educational level, residential area, number of people living together, number of children, employment, status as HCW, and the status of living with vulnerable people), health status (status of chronic medical illness, depression, and anxiety), family dynamics, and spirituality. All independent variables that are significant (*p* < 0.05) in bivariate analyses were included in the model. Multiple forward stepwise linear regression analyses were performed to investigate variables that could be the contributing factors for overall suicidal behaviour. Two-tailed *p*-value and 95% confidence intervals (95% CI) were provided. Statistical analyses were performed using IBM SPSS version 26.

## Results

The study sample included 963 participants; the majority were women (578; 60.0%) and a small proportion refused to declare their sex identity or chose “other” (74; 7.7%). The mean age of the participants was 40.1 ± 12.0 years, and about two-thirds (662; 68.7%) were between 22 and 45 years old. The majority (725; 75.3%) had tertiary education such as bachelor, master, or doctoral degree. Participants came from all types of residential areas, from rural areas (156; 16.2%) to towns (265; 27.5%) and city centres (517; 53.7%). Most of the participants were living with at least someone (916; 95.1%), less than one-third had no children (298; 30.9%), and about one-third were living with someone vulnerable (311; 32.3%). In terms of employment, about one-third were working in the public sector (357; 37.1%), and 129 (13.4%) of the participants were college or university students. The majority (817; 84.8%) did not work in the health sector. Table 1 shows further details of the sociodemographic background of the participants.

**TABLE 1:** Background sociodemography of the participants.

Sociodemographic factors		*N* (%)
Sex	Female	578 (60.0)
	Male	311 (32.3)
	Other/do not want to declare	74 (7.7)
Age (years old)	≤ 21	27 (2.8)
	22–45	662 (68.7)
	46–60	200 (20.8)
	≥ 61	74 (7.7)
Marital status	Single	232 (24.1)
	Married (or in a civil partnership)	618 (64.2)
	Divorced (or estranged)	51 (5.3)
	Co-habitant	48 (5.0)
	Widower	12 (1.2)
	Other	2 (0.2)
Educational level	Elementary school or less	35 (3.6)
	High school degree or equivalent	176 (18.3)
	Bachelor’s degree	419 (43.5)
	Master’s degree	247 (25.6)
	Doctorate (Ph.D.)	59 (6.1)
Residential area	Capital city	279 (29.0)
	City > 1 million population	147 (15.3)
	City (100,000–1 million population)	91 (9.4)
	Town (20,000–100,000 inhabitants)	108 (11.2)
	Town (< 20,000 inhabitants)	157 (16.3)
	Rural area – village	156 (16.2)
	Other	25 (2.6)
Number of people living together	1	47 (4.9)
	2	140 (14.5)
	3	265 (27.5)
	4	231 (24.0)
	5 and more	280 (29.1)
Number of children	0	298 (30.9)
	1	128 (13.3)
	2	239 (24.8)
	3	147 (15.3)
	4 or more	151 (15.7)
Living with vulnerable people	No	652 (67.7)
	Yes	311 (32.3)
Employment	Working at the public sector	357 (37.1)
	Working at private sector	192 (19.9)
	Self-employed	127 (13.2)
	Retired	64 (6.6)
	Unemployed	16 (1.7)
	Housekeeper	34 (3.5)
	College or university students	129 (13.4)
	Not working by choice	9 (0.9)
	Other	35 (3.6)
Status as healthcare workers (HCW)	Doctor	55 (5.7)
	Nurse	25 (2.6)
	Other HCW with direct contact to clinical work	31 (3.2)
	Administrative employee at a hospital	27 (2.8)
	Other hospital staff	8 (0.8)
	Do not work in the health sector	817 (84.8)

Of the total participants, 180 (18.7%) had chronic medical diseases as tabulated in Table 1. For depression, the mean ± SD of CESD scores was 21 ± 10.51 and the scores ranged from 3 to 56. For anxiety, the mean ± SD of STAI scores was 45.92 ± 9.60 and the scores ranged from 20 to 78. The mean ± SD for the total score of family dynamic parameters was −0.59 ± 3.70 and the scores ranged from −12.00 to 11.00. The spirituality score ranged from 0 to 3, with a mean ± SD of 1.74 ± 1.05.

### Bivariate analyses of the associations between suicidal behaviours and the possible contributing factors

Table 2 summarises the bivariate analyses of the associations between suicidal behaviours and the possible contributing factors. The tests showed that overall suicidal behaviours were associated with sex (*F* = 10.278, *p* < 0.001), marital status (*F* = 8.074, *p* < 0.001), educational level (*F* = 3.567, *p* = 0.003), employment type (*F* = 8.747, *p* < 0.001), residential area (*F* = 5.481, *p* < 0.001), number of people living together (*F* = 4.048, *p* = 0.003), number of children (*F* = 2.556, *p* = 0.038), depression (*r* = 0.440, *p* < 0.001), anxiety (*r* = 0.311, *p* < 0.001), and family dynamic (*r* = –0.088, *p* = 0.009). Overall suicidal behaviours and each domain of RASS (intention, lifetime, and history) were associated with sex, employment type, educational level, number of people living together, number of children, anxiety, and depression. On the contrary, none of the suicidal behaviour domains was associated with the existence of chronic medical diseases.

**TABLE 2:** The bivariate analyses of associations between suicidal behaviours and the possible contributing factors.

	Overall suicidal behaviour		Suicidal intention		Lifetime suicidal behaviour		History of suicidal behaviour	
Possible contributing factors	RASS total	*P*-value	RASS intention	*P-*value	RASS life	*P*-value	RASS history	*P-*value
Sex	10.278	0.000*	3.020	0.049*	17.108	0.000*	18.235	0.000*
Age	0.779	0.506	2.382	0.068	2.236	0.083	3.140	0.025*
Marital status	8.074	0.000*	9.864	0.000*	0.991	0.422	3.888	0.002*
Educational level	3.567	0.003*	5.268	0.000*	7.678	0.000*	6.048	0.000*
Job								
Employment type	8.747	0.000*	8.522	0.000*	8.380	0.000*	6.714	0.000*
Status as healthcare workers (HCW)	2.107	0.062	3.951	0.001*	2.248	0.048*	4.619	0.000*
Living arrangement:								
Residential area	5.481	0.000*	11.202	0.000*	1.269	0.269	4.625	0.000*
Number of people living together	4.048	0.003*	15.221	0.000*	13.251	0.000*	2.632	0.033*
Number of children	2.556	0.038*	5.983	0.000*	8.285	0.000*	4.938	0.001*
Living with vulnerable people^a^	0.884	0.990	2.489	0.077	–3.187	0.000*	2.197	0.867
Health status								
Chronic medical illness^a^	–0.799	0.425	–0.751	0.453	–0.886	0.376	0.330	0.741
Depression^b^	0.440	0.000*	0.615	0.000*	–0.123	0.000*	0.297	0.000*
Anxiety^b^	0.311	0.000*	0.353	0.000*	–0.070	0.000*	0.382	0.000*
Family dynamic^b^	–0.088	0.009*	–0.298	0.000*	0.242	0.000*	0.011	0.736
Spirituality^b^	–0.020	0.526	–0.115	0.000*	0.106	0.001*	0.038	0.235

*p < 0.05.

^a^Independent *T*-test was used for analyses between suicidal behaviour and sociodemographic factors with two categories; status of living with vulnerable people and status of having a chronic medical disease. ^b^Pearson’s correlation was used to test the association between total scores of each suicidal behaviour parameters and total score of CESD for depression, total score of STAI for anxiety, total score of family dynamic and total score of spirituality. One-way ANOVA test was used for other inferential analyses between each suicidal behaviour parameters and independent variables with three or more categories. *P*-value is the test which is significant with *p* < 0.05.

### Factors contributing to suicidal behaviour

Table 3 summarises the multivariate forward linear regressions of three proposed models. All three models are statistically significant with low adjusted *R*^2^ and low collinearity scores. The first model includes depression, sex, family dynamic, and the number of people living together as the significant contributing factors to suicidal behaviours. The second model includes the number of children as another additional factor (a protective factor). The third model shows that depression, sex, family dynamics, number of people living together, the number of children, and marital status changes are significant contributing factors to suicidal behaviours.

**TABLE 3:** Proposed models of factors contributing to suicidal behaviour.

	Model	Adjusted *R*^2^	Adjusted beta	*T*	*P*-value	95.0% confidence interval for B		Collinearity statistics	
						Lower bound	Upper bound	Tolerance	VIF
1	(Constant)	0.192		4.954	0.000	1.960	4.532		
	Depression		0.456	13.286	0.000	0.176	0.237	0.801	1.249
	Sex		0.118	3.822	0.000	0.413	1.284	0.991	1.009
	Family dynamic		0.078	2.273	0.023	0.013	0.183	0.800	1.250
	Number of people living together		0.070	2.170	0.030	0.028	0.551	0.897	1.115
2	(Constant)	0.201		4.929	0.000	1.932	4.489		
	Depression		0.459	13.446	0.000	0.177	0.238	0.800	1.250
	Sex		0.109	3.542	0.000	0.350	1.219	0.984	1.016
	Family dynamic		0.071	2.074	0.038	0.005	0.174	0.797	1.255
	Number of people living together		0.120	3.387	0.001	0.208	0.781	0.739	1.353
	Number of Children		–0.113	–3.350	0.001	–0.602	–0.157	0.814	1.228
3	(Constant)	0.205		2.896	0.004	0.729	3.793		
	Depression		0.458	13.443	0.000	0.177	0.237	0.800	1.250
	Sex		0.099	3.205	0.001	0.277	1.153	0.965	1.037
	Family dynamic		0.070	2.044	0.041	0.003	0.172	0.797	1.255
	Number of people living together		0.140	3.824	0.000	0.279	0.869	0.696	1.436
	Number of children		–0.140	–3.908	0.000	–0.706	–0.234	0.719	1.391
	Marital status		0.072	2.198	0.028	0.046	0.821	0.861	1.161

## Discussion

Due to MCO during the COVID-19 pandemic, psychological effects were evident among the general population. Suicidal behaviour was one of the main outcomes of this study. We found that suicidal behaviour was highly associated with sex, marital status, educational level, employment type, residential area, number of people living together, number of children, and family dynamics. Most participants in this study were educated; therefore, they had better access to the internet and participated in this study. WHO found women to have a higher risk of suicidal behaviour, although they have lower rates of suicide compared to men. Knowledge of the importance of gender factors shows the importance of paying attention to each gender when suicidal behaviour is identified.

A straightforward explanation may be that women expressed suicidal behaviour more than men, probably due to higher emotional sensitivity toward stress, especially during adverse life events (18). A few other local studies found a higher prevalence of depression and anxiety among Malaysian women (19) and a higher rate of suicidal ideation among female HCW during the MCO (14).

According to a systematic review by Mamun (1), loneliness and social isolation caused by MCO during COVID-19 affected more those who are alone, such as those divorced, separated, widows, or those with no children or staying alone. These findings matched our findings that these sociodemographic factors had a higher risk of suicidal behaviour during the pandemic. Our study found that higher educational status was linked to suicidal behaviour during the pandemic. This could be explained by the fact that high educational attainment leads to stability in employment, and the pandemic causes a sudden loss of jobs, hence causing high frustration and distress. This is in line with other studies that found that a high educational level was associated with a higher risk of suicidal behaviours (20, 21). Those with unemployment were found to be associated with suicidal behaviours, and financial constraints may precipitate economic stunting during the pandemic (22).

This study found that suicidal behaviour during the COVID-19 pandemic was associated with depression and anxiety. This finding was similar to other studies done during the same time (1, 8, 10, 11). This finding was expected, as suicidal behaviour could be a manifestation of depression and anxiety and also the consequence of these psychological problems. Depression and anxiety are known risk factors for suicidal behaviour, and during the COVID-19 pandemic, people may develop depressive symptoms and anxiety following reports of deaths, increased media communications, and an escalating number of new cases (23). Another study found an association between depressive symptoms, COVID-19 preventive practice measures, daily activities in home quarantine, and suicidal behaviours (24). Another review of sociocultural risk and predisposing factors for suicidal behaviour in developing countries revealed that the fear of being infected with COVID-19, growing economic pressure, and lack of resources due to lockdown were significant (25).

Surprisingly, in our study, having a chronic medical illness was not a significant contributing factor to suicidal behaviour. This is contrary to findings by other studies, which demonstrated that the presence of comorbid medical illnesses like diabetes, cerebrovascular diseases, heart diseases, and other chronic conditions would increase the risk of developing mental health problems, including suicidal behaviour (9–11, 26, 27). We were unable to demonstrate the association between suicidal behaviour and having chronic medical illness because this study did not take into account the severity and types of the chronic medical illness. Another explanation was that a previous study showed the effect that medical illness has on a person’s life in terms of disruption to daily activity rather than the number of medical conditions that predict suicide risk (28), which could explain the lack of association in our study.

However, from an ethnocultural point of view, illness perception differs between different religions in Malaysia. Malay Muslims believed suffering and diseases were trials from God for a better life in the everlasting world, while Chinese Taoists perceived illnesses to be an imbalance of forces in the body system. On the contrary, Christians may believe illnesses are due to personal sins and are a form of cleansing (29).

There were some practical strategies for reducing and managing suicidal behaviours during the COVID-19 pandemic. The government needs to address COVID-19-related unemployment and financial insecurity through financial provisions like tax deferral, wage subsidy, and investment in the labour market programme, as well as support for employers, to help them retain their workers (30). In Malaysia, the government used fiscal policy to allocate a huge budget from the lowest income individuals to the highest international trade to reduce the economic implications caused by the outbreak of COVID-19 (31). Other suicide prevention strategies include improving access to mental healthcare; responsible media reporting with information about available support; preventing increased alcohol intake; and limiting access to lethal means of suicide (32).

## Limitations and recommendations

Since this was a cross-sectional study, we were unable to demonstrate a causal-effect relationship. The data collection was fully online and distributed *via* social media, therefore this study was limited to participants with internet access and social media. There was also selection bias due to the convenience sampling method, which may affect the generalizability of the results to the general population. There were also other confounding factors not studied, like the history of suicidal attempts, life events, and family history of suicide. We would like to recommend a future prospective study to investigate this topic in more detail, to include other factors that could influence suicidal behaviours using randomised sampling and both online and physical data collection.

## Conclusion

This study revealed that depression, sex, family dynamics, the number of people living together, the number of children, and marital status are significant contributing factors to suicidal behaviours. With an understanding of the related variables associated with suicidal behaviours among the general population, which is supported by data, future mental health support can be provided for intervention, prioritising the at-risk group. This will also help in future preparedness for an unforeseen pandemic.

## Data availability statement

The raw data supporting the conclusions of this article will be made available by the authors, without undue reservation.

## Ethics statement

The studies involving human participants were reviewed and approved by the Research Ethics Committee Research Management Centre Universiti Teknologi MARA. The patients/participants provided their written informed consent to participate in this study.

## Author contributions

SR, JS, NH, and NR were the primary authors in analyzing and writing the manuscript. DT, DS, and KF were involved in the design and conceptualization of the whole research project. All authors contributed to the article and approved the submitted version.
